# Digital twin simulations with a micro-multiphysics agent-based model reveal key drivers of bone loss after denosumab discontinuation

**DOI:** 10.3389/fbioe.2025.1652201

**Published:** 2025-12-01

**Authors:** Charles Ledoux, Jack J. Kendall, Daniele Boaretti, Ralph Müller, Caitlyn J. Collins

**Affiliations:** 1 Institute for Biomechanics, ETH Zurich, Zurich, Switzerland; 2 Department of Biomedical Engineering and Mechanics, Virginia Tech, Blacksburg, VA, United States

**Keywords:** digital twin simulations, micro-multiphysics agent-based model, bone loss, denosumab discontinuation, post-menopausal osteoporosis

## Abstract

**Background:**

Denosumab is a widely used pharmacological treatment for osteoporosis-related bone fragility; however, its discontinuation is followed by a rapid drop in bone density.

**Methods:**

We investigate proposed mechanistic hypotheses from literature for this rapid bone loss using a computational micro-multiphysics agent-based model validated against clinical data. Using a representative selection of iliac crest patient biopsies scanned with micro-computed tomography, this model generates digital twin simulations of denosumab discontinuation after various treatment periods, with ceteris paribus implementations of each mechanistic hypothesis.

**Results:**

Our mixed effects linear regression analysis suggests that only the gate-blocking effect (p=0.014) and osteomorphs recycling (p=0.007) explain the rapid bone loss post denosumab discontinuation. In silico cell and cytokine dynamics emphasize that fusion of osteomorphs is more rapid than osteoclast precursor differentiation in the short-term.

**Conclusion:**

These findings highlight potential targets for managing fracture risk when discontinuing denosumab and emphasize the importance of personalized treatment strategies based on high-resolution imaging in addition to bone turnover marker measurements.

## Introduction

Global life expectancy increased from 64.9 years in 1995 to 73.3 years in 2024 (World population prospects, 2024). This has led to a rise in the prevalence of age-related osteoporosis, which is associated with an increased risk of bone fracture (Bouxsein et al., 2010). As a direct result of such hip or spine fragility fractures, an estimated 250,000 deaths occurred in the European Union, Switzerland and the United Kingdom in 2019 (Kanis et al., 2018), and at least 400,000 deaths worldwide (Shen et al., 2022).

International clinical guidelines recommend regular evaluations of bone mineral density (BMD) in women over the age of 65, men over the age of 70, individuals with prior fragility fractures, individuals over the age of 50 with risk factors to assess fracture risk and prescribe treatments for individuals at high risk of fracture with pharmacologic agents (Shoback et al., 2020; Lim et al., 2009; ACOG Committee on Clinical Practice Guidelines–Gynecology, 2021, 2022; Viswanathan et al., 2018). Such treatments include: anti-catabolic treatments, namely bisphosphonates, denosumab, and selective estrogen receptor modulators (SERMs); the dual-action drug romosozumab; and anabolic PTH analogs (LeBoff et al., 2022). The most widely used pharmacological treatment option remains bisphosphonates but these have only been demonstrated to build bone for up to 3 years of treatment (Sanderson et al., 2016). The RANKL-antibody denosumab is the only pharmacological option that has been shown to lead to a continuous increase in BMD for as long as 10 years (Dempster et al., 2018); however, the longer patients are treated with denosumab the higher the risk of atypical femoral fractures or osteonecrosis of the jaw and the larger the drop in bone density after discontinuation of denosumab.

A major concern with denosumab therapy is the rapid and large drop in BMD upon discontinuation of treatment (Bone Bolognese et al., 2011). The approved dosage for denosumab is a subcutaneous injection of 60 mg every 6 months. Unless denosumab is followed by another treatment option, a large increase in bone turnover markers (BTMs) is observed approximately 6–8 months after the last dose of denosumab. Specifically, bone resorption markers (e.g., CTX, NTX) become significantly higher relative to baseline than bone formation markers (P1NP) (Miller et al., 2008). This imbalance results in a decrease in BMD to below baseline levels. This rapid drop in BMD is associated with increased risk of fracture in an already at-risk population (Cummings et al., 2018). Moreover, the elevated bone turnover and steep rate of bone loss result in a return to pre-treatment BMD levels approximately 18 months after the last denosumab dose (Zanchetta et al., 2018; Venkataraman et al., 2017; McClung et al., 2017).

To improve clinical outcomes for patients with osteoporosis, more clinical trials in the past decade than ever before have focused on drug sequencing (Miller et al., 2008; Guañabens et al., 2019; Takeda et al., 2012; Bone et al., 2018), combining drugs (Leder, 2018; Schafer et al., 2012; Finkelstein et al., 2010; Hejdova et al., 2005) and improving patient adherence (compliance) to a drug regimen (Inderjeeth et al., 2014). However, phase 3 clinical trials cost on average 30 million USD over the course of 1–4 years (May, 2019); this cost increases by an estimated 671,000 USD with each additional month (Martin et al., 2017), meaning that trials on osteoporosis medication lasting up to 10 years are particularly expensive. Recently, *in silico* simulations have been developed that can provide fast, inexpensive and ethical alternatives to years of costly experimentation on animals and humans (Tourolle David Dempster et al., 2021; Martínez-Reina and Pivonka, 2019; Martínez-Reina et al., 2021a; Martínez-Reina et al., 2021b; Martínez-Reina et al., 2009; Buenzli et al., 2012; Kendall et al., 2023; Boaretti et al., 2023). These *in silico* models aim to become tools to test hypotheses on bone remodeling, for informing bone health clinical trial design, and for reflecting performance of osteoporosis drugs when patients are not adherent to prescribed doses. As of 2024, patient compliance with the approved treatment options remains the biggest detriment to treatment outcomes (McCloskey et al., 2021) and there are no new drugs for osteoporosis on the horizon because treatment regimens have failed to get past phase 2 clinical trials (Recker et al., 2020).

Existing *in silico* models have been developed to study bone remodeling, its dysregulation during metabolic bone diseases and the effect of therapeutic- and exercise-based interventions. Within the varied spectrum of *in silico* modelling techniques, only bone cell population dynamics models and micro-multiphysics agent-based (micro-MPA) models explicitly incorporate the complex cellular and molecular mechanisms linked to metabolic bone diseases and the pathways involved in their treatments. To date, only micro-MPA models have the spatial resolution to predict the effect of the complex cell-cytokine pathways on the bone microarchitecture (Ledoux et al., 2022; Boaretti et al., 2022). Micro-MPA models represent cells as independent agents that sense their local environment and are able to modify it, leading to the emergence of complex patterns at the local and global level as observed in clinical patient data.

**FIGURE 1:**
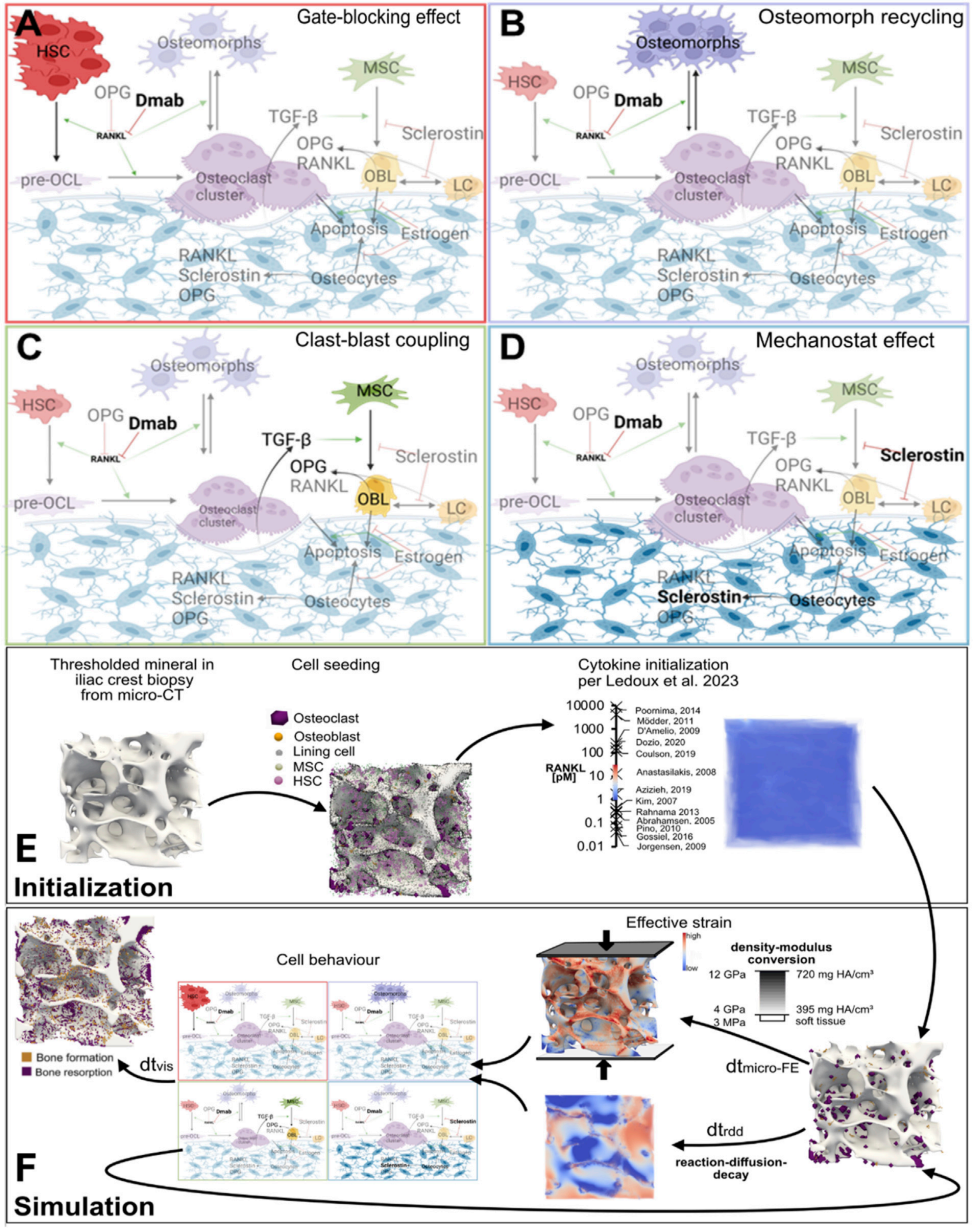
Schematics of the major cell types and biochemical signaling pathways involved in four mechanistic hypotheses for rapid bone loss following denosumab discontinuation, and a simulation workflow diagram of the micro-multiphysics agent-based model. **(A)** Gate-blocking effect: Denosumab lowers RANKL levels, blocking the differentiation of osteoclast precursors and leading to their accumulation. Upon discontinuation, this reservoir differentiates en masse into osteoclasts, triggering rapid bone loss. **(B)** Osteomorph recycling: During treatment, osteoclasts fission into osteomorphs, allowing them to persist in the marrow under low RANKL conditions. After drug clearance, rising RANKL levels drive their re-fusion into osteoclasts and reseeding onto the bone surface, leading to a spike in bone resorption. **(C)** Clast-blast coupling: Reduced osteoclast activity during treatment impairs clast-blast coupling, decreasing osteoblast numbers and OPG production. The resulting imbalance in the RANKL/OPG pathway enhances resorption after discontinuation. **(D)** Mechanostat effect: Osteocytes, sensitive to mechanical cues, increase sclerostin production after denosumab withdrawal in an attempt to restore structural mechanics to pretreatment conditions, thereby accelerating bone turnover. **(E,F)** Simulation workflow diagram: 3D visualizations of virtual biopsies at key steps of model initialization and simulation. These steps include: input micro-CT scans of iliac crest biopsies; **(E)** initialization of cell populations and cytokine fields displaying on the left one of the iliac crest biopsy µCT scans that serves as input to the model with a threshold applied to display the trabecular bone, in the middle the same scan with the initial distribution of osteoclasts, osteoblasts and lining cells on the bone surface and MSCs and HSCs in the marrow, and on the right the same scan but displaying the initial RANKL concentration at each voxel in pM along with the range of values for RANKL concentration measured in postmenopausal women reported in the literature with the associated reference for each value; and **(F)** iterative updates of the three multiphysics processes–cytokine reaction-diffusion-decay, cell behavior (based on mechanistic hypotheses A–D), and tissue mechanics. Boundary conditions for the micro-FE simulation: A uniaxial compressive displacement of 1% strain was applied to the top surface of the VOI, with the bottom surface fixed and lateral surfaces traction-free. This loading approximates physiological compression in trabecular bone under body weight. Cell types: HSC–haematopoietic stem cell, pre-OCL–surface unfused preosteoclast, osteoclasts, osteomorphs, MSC–mesenchymal stem cell, OBL–osteoblast, LC–lining cell, osteocytes. Signaling molecules: Dmab–Denosumab, OPG–osteoprotegerin, RANKL–receptor activator of nuclear factor κB ligand, TGF-β–Transforming Growth Factor β, sclerostin. Red T-bars represent inhibition, green arrows indicate activation, and black arrows display cell behaviour steps. Figures **(A–D)** created with BioRender.

Given the complexities of cell-cytokine interactions during bone remodeling and their response to treatment, probing the cellular mechanistic hypotheses responsible for the rapid bone loss after denosumab discontinuation utilizing a micro-MPA model may provide valuable insight into future clinical trials targeting the development of new treatments and drug sequencing strategies. To date, four mechanistic hypotheses (see Figures 1A–D) have been proposed to explain the rapid drop in BMD upon denosumab discontinuation: accumulation of osteoclast precursors due to blocked differentiation to osteoclasts (McClung et al., 2017), osteoclast recycling via osteomorphs (McDonald et al., 2021), a lowering of OPG levels during treatment with denosumab due to clast-blast coupling (Lacey et al., 2012; Azizieh et al., 2019; LaCroix et al., 2013; El-Masri et al., 2024) and finally conventional mechanosensitive signaling by osteocytes whereby faster evacuation of denosumab would be the only difference to bisphosphonates (Mulvihill et al., 2008). Identifying the relative contribution of each mechanism is key to creating a physiological model of denosumab treatment and discontinuation. Such a physiological model could then be used to identify therapeutic targets to prevent or mitigate bone loss as well as predict the optimal follow-up therapy for each patient (Zanchetta et al., 2018).

This study builds on a previously validated micro-MPA model simulating osteoporosis and treatment in micro-computed tomography (µCT) scans of iliac crest biopsies simulating 10 years of bone volume fraction data, as well as dynamic morphometric parameters from the FREEDOM trial (Tourolle David Dempster et al., 2021). In the current work we extend this *in silico* model to simulate denosumab discontinuation and use a mixed effects linear model analysis to investigate the relative contributions of the four mechanistic hypotheses outlined above to the bone mass accrual (treatment) and the subsequent rapid bone loss (discontinuation). Furthermore, we investigate the role *in silico* modeling and image-based sample-dependent biomarkers can have on assessing individualized patient response to both denosumab treatment and its discontinuation.

This study had two main goals: firstly, to update an existing model of denosumab treatment to accurately reflect the trends in denosumab discontinuation, and secondly, to investigate which of several literature-proposed mechanistic hypotheses contribute to the rapid bone loss after denosumab discontinuation. Micro-MPA model refinement was performed utilizing data from a systematic review of clinical trial literature (Ledoux et al., 2022) to ensure our simulations emulate the physiological responses of post-menopausal osteoporosis, denosumab treatment, and its discontinuation. The model was deemed to be stable and effective based on the robustness of the match in BMD trends between clinical trials and *in silico* simulations with different input geometries, the ability of all cell numbers and cytokine concentrations to stay within ranges reported in literature for thousands of cell behaviour and reaction diffusion iterations, and the dynamic morphometric parameters *in silico* matching rates of bone formation and resorption that are well documented for placebo and denosumab in the FREEDOM trial. Cell-seeding, cytokine initial concentrations and cytokine dynamics were individually verified using *in vivo* cell and cytokine dynamic data pulled from clinical trial literature (Ledoux et al., 2022). Subsequently, the verified model was used to generate digital twin simulations across various denosumab treatment durations, applying ceteris paribus conditions to four proposed mechanistic hypotheses for the rapid bone loss. A mixed-effects linear regression analysis of the simulated bone mineral content (BMC) over treatment and discontinuation was conducted to quantify the contribution of each mechanistic hypothesis to the rapid bone loss following denosumab discontinuation. Lastly, with the validated exploratory model, sequencing was performed to further delineate contributions of each mechanism to treatment sequences not available in the literature.

## Results

### Osteoclast precursor accumulation and osteomorphs recycling are the most significant contributors to the rapid bone loss following denosumab discontinuation

The micro-MPA model, first described in (Tourolle, 2019), was used to simulate trabecular bone remodeling during 2 years of denosumab treatment with a two-year placebo follow-up. This in-house model is based on iterative updates to three distinct physical processes: 1) reaction-diffusion of signaling molecules and binding sites, 2) cell behavior (including resorption and formation, production of signaling molecules by cells, cell apoptosis, proliferation and motion) and 3) calculation of strain distribution based on the newly remodeled microstructure. The initialization and iterative multiphysics simulation steps are depicted in Figures 1E,F. The micro-MPA model was set up using *in vivo* cell and cytokine data from the literature (Ledoux et al., 2022).

A mixed effects linear model, the details of which can be found in the methods section at the end of this manuscript, was used to quantify the contribution of each of the four possible mechanistic hypotheses for the rapid bone loss following denosumab discontinuation. As input to the mixed effects linear model, the configurations of the model α to π in Table 1 were run corresponding to on-off activations of the four mechanistic hypotheses leading to a total of 16 configurations of the model run on seven input biopsies each for a total of 112 simulations. The bone mineral content (BMC) output of each simulation was compared with clinical data from literature (Bone Bolognese et al., 2011) using the sum of squares of the difference between average relative changes in simulation output (N = 7 (placebo) and N = 7 (denosumab)) and average relative changes in clinical total hip BMD measurements at the eight timepoints at which BMD was measured in the clinical population [includes participants enrolled in the off-treatment phase with observed values at month 0 and the time point of interest, N = 110–128 (placebo) and N = 109–128 (denosumab)].

**TABLE 1:** Mixed linear effects model runs and correlations to clinical BMD tends. Overview of simulations for the mixed effects linear model study over 2 years of treatment followed by 2 years of discontinuation. For each simulation, theme an arctangent absolute percentage error in degrees (MAAPE°) and the coefficient of determination R^2^ are reported for the percent change in bone mineral content (BMC) compared to the percent change in clinical bone mineral density (BMD).

Model	Gate-blocking effect	Osteomorph recycling	Clast-blast coupling	Mechanostat effect	MAAPE°	R^2^_all_	R^2^_dmab_	R^2^_dis_	R^2^_end_
Run α	0	0	0	0	42.8				
Run β	1	0	0	0	33.3				
Run γ	0	1	0	0	31.7				
Run δ	0	0	1	0	30.4				
Run ε	0	0	0	1	30.1				
Run ζ	1	1	0	0	18.7				
Run η	1	0	1	0	28.0				
Run θ	1	0	0	1	33.4				
Run ι	0	1	1	0	15.7				
Run κ	0	1	0	1	27.0				
Run λ	0	0	1	1	35.1				
Run μ	1	1	1	0	15.6				
Run ν	1	1	0	1	17.9				
Run ξ	1	0	1	1	38.5				
Run ο	0	1	1	1	37.7				
Run π	1	1	1	1	11.2				
p-value mixed effects model	**0.014**	**0.007**	0.159	0.090					

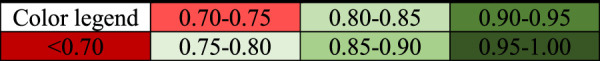

R^2^_all_: entire timeline from baseline to month 48. R^2^_dmab_: treatment phase from baseline to month 24. R^2^_dis_: phase immediately following discontinuation from month 24 to month 36. R^2^_post_: progressive stabilization in the post-discontinuation phase from month 36 to month 48. Bold values indicates statistical significance (p<0.05).

Coefficient of determination R^2^ is reported for each simulation in Table 1, reflecting the degree to which the percent change in BMC in the simulation predicts *in vivo* percent change in BMD from baseline to the completion of the study ( $R_{all}^{2}$ ), during the treatment phase ( $R_{dmab}^{2}$ ), immediately following discontinuation ( $R_{dis}^{2}$ ), and at late stage discontinuation ( $R_{end}^{2}$ ). To evaluate model performance, we computed not only the coefficient of determination (R^2^) but also the Mean Arctangent Absolute Percentage Error in degrees (MAAPE°) between simulated percentage change in BMC and clinical percentage change in BMD. MAAPE° provides a robust notion of relative error that remains bounded and stable when percentage changes approach zero, complementing R^2^ in settings with low variance or skewed trends (Kim and Kim, 2016). While BMD showed consistently low MAAPE° (<30°) and high R^2^ (>0.8) across most configurations, CTX and P1NP displayed higher relative error and variability. Over all time periods, the coefficient of determination is highest for model configurations with both the gate-blocking effect and osteomorphs recycling activated. If these are activated, the model performs well regardless of whether clast-blast coupling and the mechanostat effect are activated. The regression analysis over all configurations α to π of the model over all time periods confirms that the gate-blocking effect (p = 0.014) and osteomorphs recycling (p = 0.007) are significant contributors to the clinically observed changes in bone mineral during treatment and discontinuation.

### All four mechanistic hypotheses in isolation qualitatively follow clinical changes in bone density, formation and resorption during treatment and discontinuation

**FIGURE 2:**
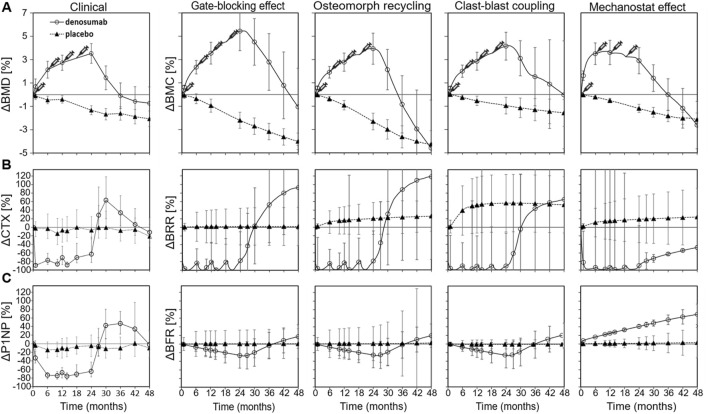
Comparison between clinical data and virtual biopsy outcomes for four mechanistic hypotheses for the rapid bone loss following denosumab discontinuation. **(A)** Percent change in clinical bone mineral density (BMD) (left) and in the bone mineral content (BMC) of virtual biopsies (right) **(B)** Percent change in the bone resorption blood biomarker carboxy-terminal collagen crosslinks (CTX) (left) and in the *in silico* bone resorption rate (BRR) (right) **(C)** Percent change in the bone formation blood biomarker procollagen type 1 propeptide (P1NP) (left) and in the *in silico* bone formation rate (BFR) (right). Error bars represent standard errors. Syringes indicate the timing of subcutaneous denosumab injections. For each simulation configuration, the clinical data are provided in the background in grey.

Relative changes in bone mineral content (BMC) from simulation runs β to ε qualitatively matched clinical trends in bone mineral density (BMD), as shown in Figure 2A. These runs represent isolated implementations of the gate-blocking effect, osteomorph recycling, clast-blast coupling, and mechanostat regulation with each being selected as the best-fitting version of their respective mechanistic hypothesis. In all four cases, BMC increases during the denosumab treatment period and then declines rapidly, with onset between 6 and 9 months after the final injection. While average BMC falls below baseline in all cases, only in the osteomorph recycling scenario does it decline below the placebo curve. Among the four, the mechanostat model displays the most divergent behavior from the clinical data, with a too rapid initial rise in BMD and a relative plateau between 6 and 18 months during treatment. This is because achieving rapid post-treatment bone loss in this scenario (so high BRR from months 24–36) is very difficult and requires a prolonged period during which osteocytes are stimulated to produce sclerostin. This leads to bone resorption returning to baseline levels after denosumab discontinuation, while bone formation remains suppressed.

Relative changes in bone resorption rate (BRR) and bone formation rate (BFR) for simulation runs β to ε are shown in Figures 2B,C, respectively, alongside corresponding clinical data derived from BTMs, specifically serum C-terminal telopeptide of type I collagen (CTX) for resorption and procollagen type I N-terminal propeptide (P1NP) for formation. Each simulation represented an isolated mechanistic hypothesis, including the gate-blocking effect, osteomorph recycling, clast-blast coupling, and mechanostat regulation. Temporal remodeling dynamics demonstrated a qualitatively close alignment with clinical BTM trends. In both the clinical trial data and the iliac crest micro-MPA simulations, BRR increases sharply around 6 months after the final denosumab injection, marking the onset of rebound bone loss. This resorptive peak is followed by a gradual stabilization of both BRR and BFR to levels below baseline, though still elevated relative to the placebo group by month 48. These findings suggest that the mechanisms modeled in simulation runs β to ε successfully reproduce not only the short-term rebound phase but also the longer-term remodeling behavior observed clinically after denosumab discontinuation.

### The micro-MPA model facilitates a visual and quantitative analysis of the cells and signaling molecules involved in each mechanism

**FIGURE 3:**
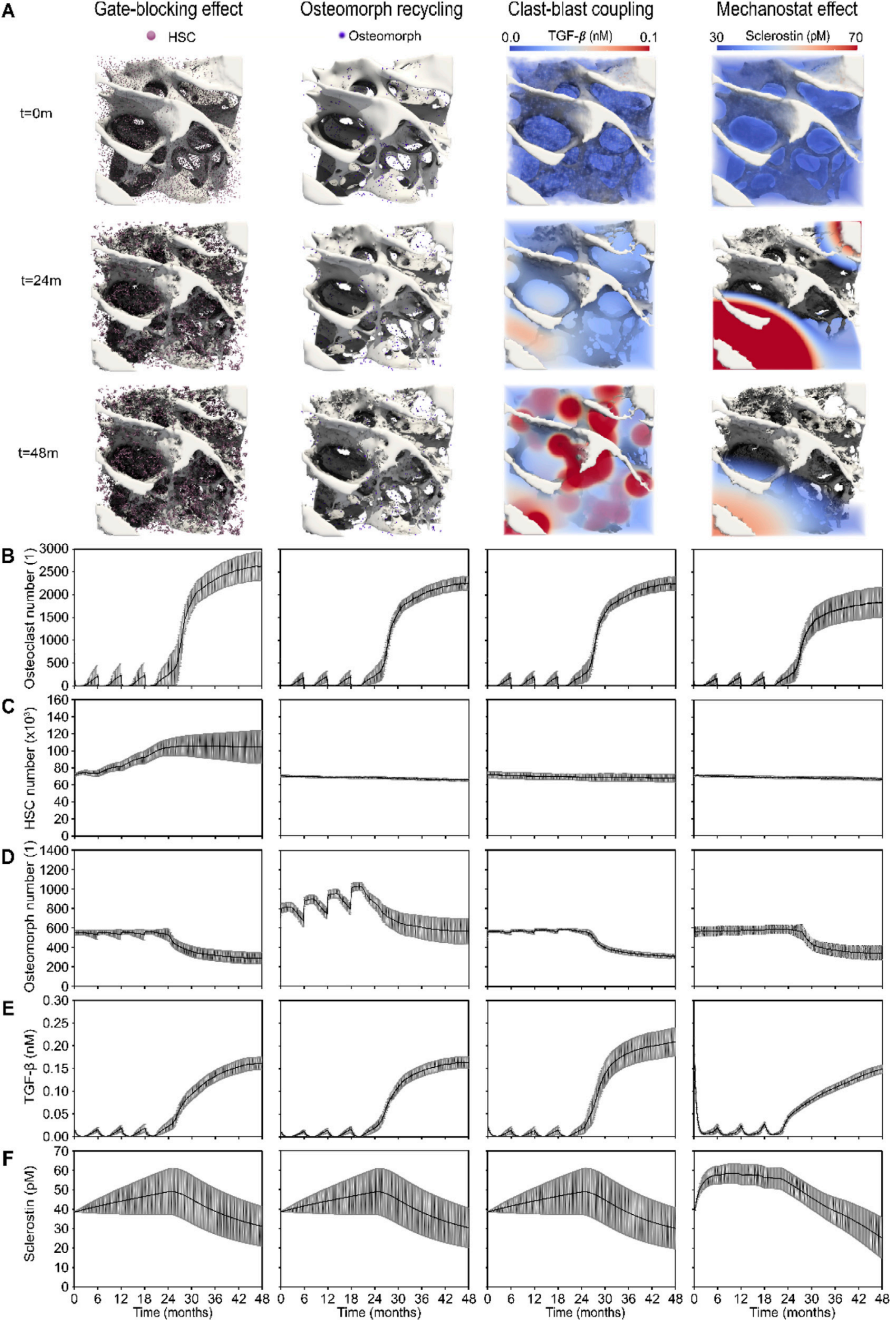
3D visualizations and quantitative analysis based on cell number and signaling molecule concentration dynamics across the four mechanistic hypotheses for the rapid bone loss following denosumab discontinuation. **(A)** Gate-blocking effect: accumulation of haematopoietic stem cells (HSCs, pink) during treatment; Osteomorph recycling: osteomorphs (purple) serving as a recycling pathway for osteoclasts as an alternative to apoptosis; Clast-blast coupling: drop and rise in the coupling signaling molecule transforming growth factor β (TGF-β); Mechanostat effect: rise and drop in the mechanostat signaling molecule sclerostin. **(B)** Osteoclast numbers, showing rapid post-injection cell death and progressive recovery in the simulations. **(C)** HSC numbers, highlighting the distinct accumulation seen in the gate-blocking effect. **(D)** Osteomorph numbers, illustrating the specific elevation in osteomorph recycling. **(E)** TGF-β concentration (nM), showing a more pronounced drop and subsequent rise in clast-blast coupling. **(F)** Sclerostin concentration (nM), showing a steeper initial rise in the mechanostat effect scenario. Solid lines represent the mean simulation results across all digital twins (N = 7); grey shaded areas denote the standard error across patients.

**FIGURE 4:**
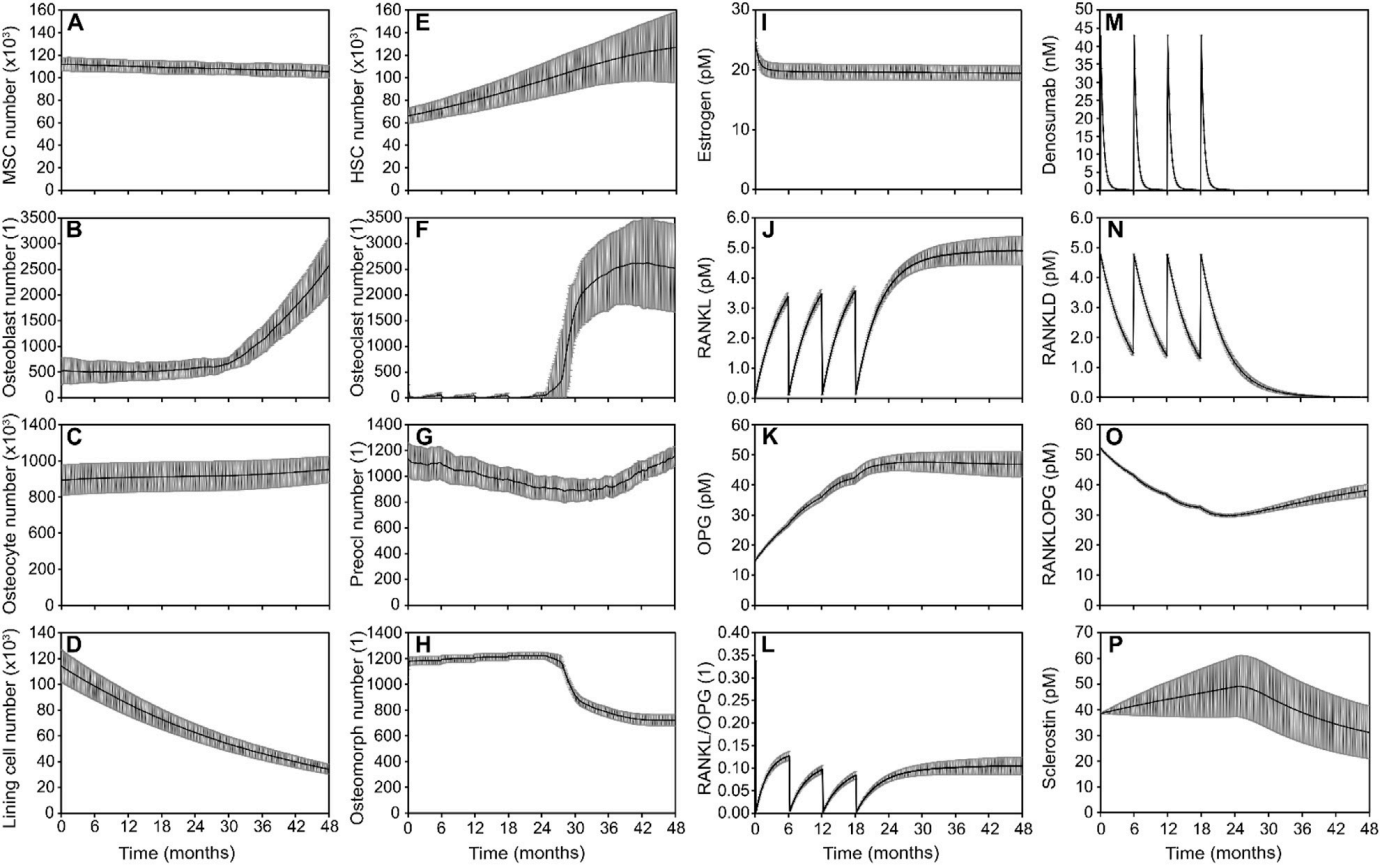
Simulated responses to denosumab discontinuation showing the combined effect of bone cell populations and signaling pathways in the optimal model. Osteoblast lineage cells: **(A)** Mesenchymal stem cell (MSC) number, **(B)** Osteoblast number, **(C)** Osteocyte number**, (D)** Lining cell number; Osteoclast lineage cells: **(E)** Hematopoietic stem cell (HSC) number **(F).** Osteoclast number **(G).** Preosteoclast number **(H).** Osteomorph number; Signaling molecule concentrations: **(I)** Estrogen concentration **(J).** RANKL concentration **(K).** OPG concentration **(L)** RANKL/OPG ratio **(M)** Denosumab concentration **(N)** RANKL-denosumab complex concentration **(O)** RANKL-OPG complex concentration **(P)** Sclerostin concentration. Solid lines represent the mean simulation results across all digital twins (N = 7); grey shaded areas denote the standard error across patients.

The simulated outcomes of the four mechanistic hypotheses proposed to explain rapid bone loss following denosumab discontinuation are illustrated through 3D visualizations (Figure 3A) and quantitative trends in cell numbers and signaling molecule concentrations (Figures 3B–F). Each simulation outcome shown in Figures 3, 4 reflects the mean behavior across seven patient-specific virtual biopsies, and the surrounding grey shaded areas represent the standard error, capturing inter-patient variability in bone remodeling and treatment response. Each hypothesis is characterized by a distinct mechanistic driver: the gate-blocking effect shows accumulation of haematopoietic stem cells (HSCs) during treatment due to blocked differentiation; osteomorph recycling involves an alternative fate for osteoclasts via recycling into osteomorphs instead of apoptosis; clast-blast coupling is driven by dynamic changes in transforming growth factor β (TGF-β); and the mechanostat effect involves regulation by sclerostin levels linked to strain-sensing osteocytes. Simulated osteoclast numbers display rapid drug-induced apoptosis followed by gradual repopulation across all hypotheses. HSCs accumulate only under the gate-blocking mechanism, while elevated osteomorph levels are uniquely observed in the recycling scenario. TGF-β concentrations show a distinct decline and rebound pattern specific to clast-blast coupling. In contrast, sclerostin levels rise sharply then decline in the mechanostat effect, reflecting the strain sensitivity of osteocyte signaling.

Notably, the 3D spatial visualizations of TGF-β and sclerostin in Figure 3A emphasize key differences in spatial signaling behavior. The TGF-β distribution closely follows zones of active resorption, yielding a broad and diffuse elevation wherever osteoclast activity is high. In contrast, sclerostin distribution exhibits sharper peaks and troughs due to the non-linear strain response of osteocytes. Since most osteocytes lie near the inflection point of the Hill curve governing sclerostin expression, only those at the extreme low or high ends of the strain spectrum contribute disproportionately to the resulting spatio-temporal distribution of sclerostin as seen in the mechanostat effect column of Figure 3A. This leads to exaggerated spatial contrasts in sclerostin levels and highlights a mechanistic distinction: TGF-β serves as a relatively uniform mediator of coupling during resorption, whereas sclerostin acts as a more localized and strain-sensitive modulator of bone formation.

### The optimized model of denosumab treatment and discontinuation that most closely matches clinical trends in bone density relies on the gate-blocking effect and osteomorphs recycling to provide the rapid bone drop after denosumab discontinuation and on clast-blast coupling to achieve a stable final phase

The optimized model of denosumab treatment and discontinuation that most closely reproduces clinical trends in bone density relies on a combination of three key mechanisms: gate-blocking and osteomorph recycling to drive the rapid bone loss after drug discontinuation, and clast-blast coupling to stabilize the system in the post-discontinuation phase beyond month 36. Simulated outputs show how bone cell populations and signaling molecules evolve over time to collectively capture the observed biphasic response to discontinuation leading to rapid bone loss between 24 and 36 months followed by a progressive return to baseline pre-treatment conditions over the period from month 36–48 (Figure 4).

HSCs (Figure 4E) accumulate progressively during treatment, consistent with the *gate-blocking effect*, in which denosumab suppresses osteoclast differentiation and leads to a buildup of undifferentiated progenitors. Following treatment cessation, a sharp decrease in osteomorph numbers (Figure 4H) corresponds to their rapid differentiation into mature osteoclasts (Figure 4F). This combination of a latent reservoir of HSCs and a surge of differentiating osteomorphs generates a rapid and substantial increase in osteoclast numbers. Osteoclasts appear first at the locations with the highest production of RANKL by osteocytes, which are also the locations with the lowest local effective strain. The higher the osteoclast recovery the larger the bone resorption rate and the drop in BMD following discontinuation. Osteoblast numbers (Figure 4B) rise gradually in response to the increased resorptive activity, a hallmark of clast-blast coupling. The eventual stabilization of osteoclast populations (Figure 4F) in the post-discontinuation phase reflects the return to a dynamic balance in bone remodeling.

In terms of signaling pathways, the RANKL/OPG axis shows reactivation after denosumab discontinuation, with increasing RANKL levels and a rising RANKL/OPG ratio (Figures 4J–L), promoting osteoclastogenesis. Denosumab and its bound complexes (Figures 4M,N) decline as expected following treatment cessation, releasing RANKL to act on available precursors. The RANKL–OPG complex (Figure 4O) serves as a reversible binding sink, sequestering free RANKL and thereby modulating its availability to promote osteoclast formation. This complex is not assigned any additional biological activity beyond its role in buffering RANKL levels. Meanwhile, sclerostin levels (Figure 4P) confirm that the *mechanostat* remains responsive to strain, but its comparatively modest variation suggests a limited role in driving the discontinuation dynamics in this scenario.

Overall, the model demonstrates that the best fit to clinical data involves a synergistic mechanism: gate-blocking primes the system by storing resorptive potential during treatment (Figure 4E), osteomorph recycling provides a rapid trigger for osteoclast resurgence after drug withdrawal (Figure 4H), and clast-blast coupling ensures long-term stabilization of bone remodeling (Figures 4B,F).

### The best predictor of individual iliac crest biopsies’ response to treatment with and discontinuation of denosumab is the structural model index

**FIGURE 5:**
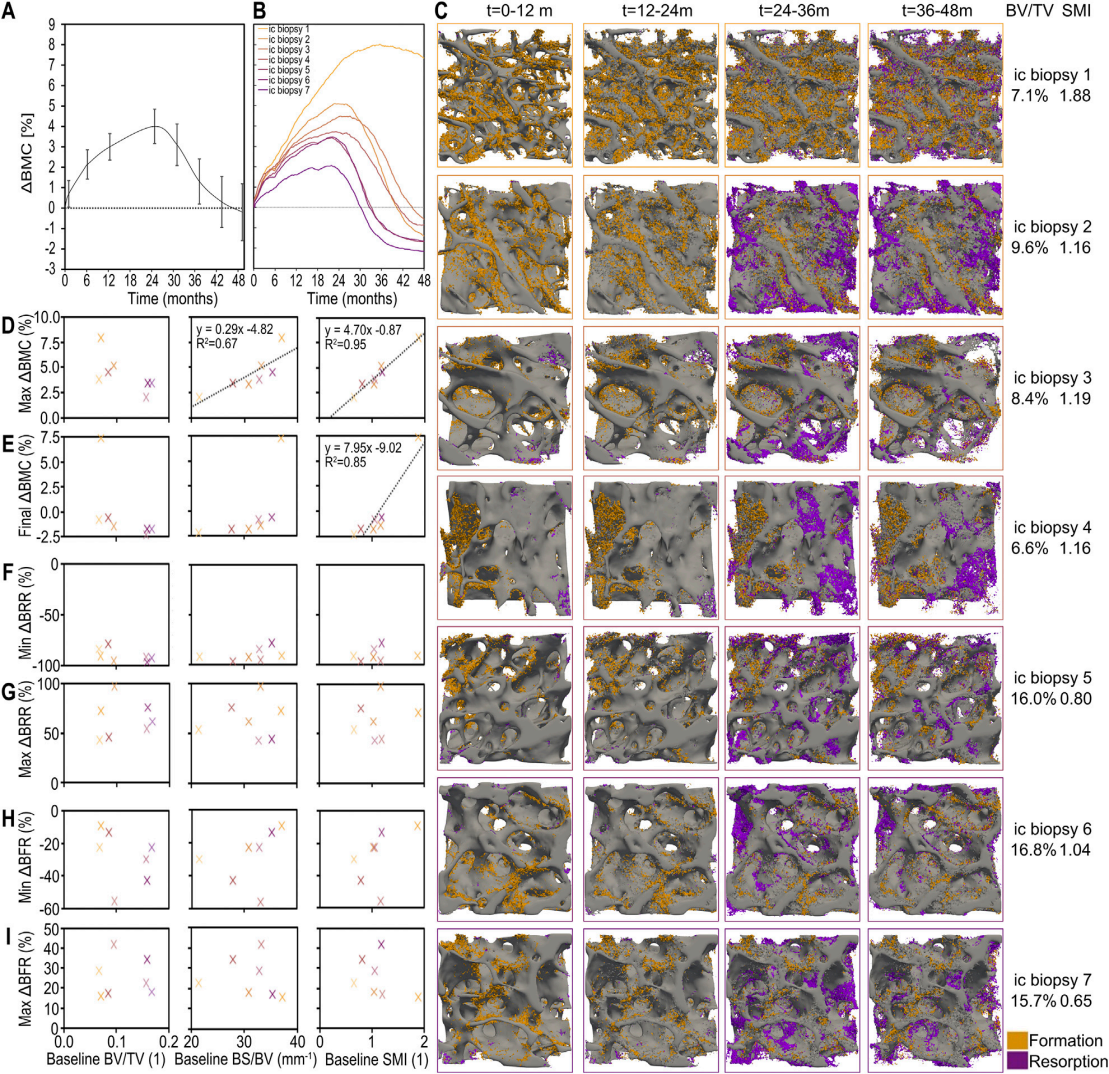
Individualized simulated responses to denosumab discontinuation showing the combined effect of changes in bone mineral content (BMC), bone formation and bone resorption, and their patient-specific relations to baseline morphometric parameters in the optimal model. **(A)** Simulated percent change in bone mineral content (BMC) across all patients (average and standard error). **(B)** Simulated patient-specific percent change in BMC. **(C)** 3D visualisations of bone formation and bone resorption for each individual iliac crest biopsy simulation. Correlation between static morphometric parameters at baseline (bone volume fraction (BV/TV), specific bone surface (BS/BV) and structural model index (SMI)) and simulated clinically relevant outcomes including: **(D)** maximum relative change in BMC; **(E)** final relative change in BMC; **(F)** minimum bone resorption rate (BRR) as a percent of baseline BRR prior to the first injection; **(G)** maximum BRR relative to baseline as a result of treatment discontinuation; **(H)** minimum bone formation rate (BFR) as a percent of baseline BFR prior to the first injection; and **(I)** maximum BFR relative to baseline as a result of treatment discontinuation. Significant correlations are displayed with black trendlines with their equations and coefficient of determination.

Figure 5 presents the outcomes of the optimized model which most closely replicates clinical bone density trends following denosumab treatment and discontinuation. Virtual patients were simulated under a two-year denosumab treatment followed by a two-year placebo phase, and the results show how changes in bone mineral content (BMC), remodeling dynamics, and baseline trabecular structure interact in this context.

The average percent change in BMC over time across all virtual biopsies is shown in Figure 5A, with standard error shading to reflect inter-individual variability. The corresponding individual BMC responses for each of the seven biopsies are shown in Figure 5B, alongside their baseline bone volume fractions (BV/TV). These results highlight the range of responses captured by the model when run on structurally diverse bone volumes.

To visualize this structural variability, Figure 5C provides 3D renderings of each biopsy’s trabecular architecture, annotated with baseline BV/TV and structural model index (SMI). When correlating baseline morphometric parameters with treatment outcomes, Figures 5D,E show that SMI is the strongest predictor of both the maximum and final relative changes in BMC. Biopsies with higher SMI, i.e. more rod-like structures, experience greater bone gain during treatment and their bone density remains higher after treatment cessation despite the resulting bone loss. This underscores the importance of trabecular geometry in determining skeletal vulnerability to bone loss after denosumab discontinuation.

In contrast, Figures 5F–I examine whether baseline structural parameters also predict the minimum and maximum bone resorption rate (BRR) and bone formation rate (BFR) relative to pretreatment levels. No significant correlations are found, suggesting that while structure determines long-term BMC outcomes, it does not directly predict short-term remodeling activity after treatment ends.

### Gate-blocking effect governs total bone loss while osteomorphs recycling explains rate of bone loss

The accumulation of HSCs in the marrow matched *in vivo* measurements more closely when osteomorphs were explicitly included as an additional cell type in the model. Specifically, the model predicted increases in osteoclast precursor numbers over the course of treatment with denosumab, in accordance with clinical data demonstrating significantly higher (p = 0.011) numbers of cells expressing CD14+/CD11b+ in a group of 15 denosumab-treated women (average age 81, median duration of treatment 4 years) compared with a group of 15 age-matched controls (Schini et al., 2024). A quantitative comparative analysis of HSC and osteomorphs cell number trends as shown in Figures 4E,H reveals that the accumulation of HSCs in the marrow over the entire 6 months interval between two injections contributes approximately twice as many resurgent osteoclasts as osteomorphs though the differentiation of osteomorphs into osteoclasts occurs faster than that of osteoclast precursors in the marrow to differentiated osteoclasts on the bone surface.

## Discussion

We have built on an existing *in silico* experimental platform (micro-MPA) for spatiotemporal observation and prediction of bone physiological and pathological conditions resulting from complex intercellular signaling. In conjunction with *in vivo* and *in vitro* experiments, *in silico* experiments provide a third avenue to explore bone metabolism and may thus accelerate research. Furthermore, we anticipate that our verified and validated micro-MPA model will prove valuable in clinical practice, such as in comprehensive drug assessment and formulation of effective treatment regimens.

We applied the micro-MPA model to predict the effects of osteoporosis, denosumab treatment and discontinuation in a cohort of postmenopausal women and demonstrated that *in silico* medication experiments provide a powerful way to assess the effects of drugs on bone cells and morphology in clinically relevant scenarios. Our *in silico* model reproduced trends in BMD observed experimentally after discontinuation of denosumab.

We have investigated *in silico* four possible mechanisms for the rapid bone loss after discontinuation of treatment with denosumab: accumulation of osteoclast precursors or osteoclast recycling via osteomorphs during treatment, imbalance in the RANKL/OPG ratio due to decreased osteoblast numbers, and increased production of sclerostin by osteocytes to reset the mechanics of the structure to pretreatment conditions (Laroche et al., 2023).

Limitations of this study include that the performance of the model with each mechanism implemented was evaluated based on a direct comparison between percentage changes in clinical BMD and percentage changes in in silico BMC, without applying a conversion between these quantities. As this approach is novel, it remains unclear whether a standardized conversion is needed. This challenge stems from the broader difficulty in validating mechanistic *in silico* models with human clinical data, particularly when longitudinal HR-pQCT scans of the same patients are unavailable. While BMC and BMD are strongly correlated, further work is required to determine whether direct comparison is appropriate across modalities or whether a regression-derived mapping function or simulation-specific calibration is necessary. Establishing such a framework would improve the reliability of model evaluation and facilitate its future use in clinical contexts.

Despite strong agreement for BMD trajectories, model predictions of BTMs (CTX, P1NP) deviated from clinical observations, especially during the rebound phase post-denosumab. This discrepancy may result from oversimplified assumptions regarding cytokine clearance and production, or the use of population-averaged initial conditions that may not capture transient peaks and dips in BTMs. Furthermore, while BMD integrates long-term structural adaptation, BTMs fluctuate rapidly with cell activity and systemic influences, making them inherently more variable and harder to fit. It is important to note that the 7 samples used to initialize the *in silico* study are from a different patient population than the population used for the clinical comparison, hence the aim to approximately predict trends rather than match results exactly.

Another important limitation of this study is the comparison between site-specific *in silico* remodeling (based on iliac crest biopsies) and systemic BTMs such as CTX and P1NP. This assumes that the iliac crest reflects average systemic trabecular remodeling activity - a common assumption in clinical research due to its accessibility and trabecular-rich nature. However, skeletal site heterogeneity is well-documented, and treatment effects may vary across sites such as the spine, femur, and tibia. Consequently, while general trends may be comparable, direct quantitative matching of iliac-based remodeling with systemic BTMs must be interpreted cautiously. Future model extensions could simulate multiple skeletal sites or use scaling models to bridge local remodeling and systemic outputs.

While the linear combination model helps to assess which mechanistic features are necessary to recapitulate clinical BMC trends, it does not fully resolve the relative strength or dominance of each mechanism. The use of binary activation variables assumes the presence or absence of a pathway, but not its graded or quantitative impact. Moreover, the interplay between mechanisms may be synergistic or antagonistic in ways that are not fully captured by the regression model, especially under conditions where certain pathways overpower others in their influence on remodeling dynamics. Further validation of the magnitude of effect associated with each pathway will require experimental perturbation data or parameter-specific sensitivity analyses targeting individual mechanistic axes.

In the current micro-MPA model, we focus on simulating the behavior and interactions of bone-resorbing and bone-forming cells in a spatially explicit environment, including mechanotransduction and cytokine signaling. While this framework captures key dynamics of bone remodeling under denosumab and placebo conditions, it does not explicitly simulate all contributors to bone mineral density (BMD) changes. In the micro-MPA model the mineralization kinetics were simplified such that at any timestep the new mineral formation in a given voxel was proportional to the difference between the osteoid and the mineral in that voxel. The duration of the primary mineralization phase was set to 1 week so that a voxel saturated with osteoid and empty of mineral would reach 70% mineralization within a week. This 1 week duration of the primary mineralization phase is at the lower end of the durations measured *in vivo* (Lukas et al., 2013; Bala et al., 2010; Ruffoni et al., 2007; Roschger et al., 2008). In addition, the simplified mineralization kinetics implemented in the micro-MPA model are missing elements such as the mineralization lag time implemented in other *in silico* studies exploring bone mineralization kinetics in more detail (Castoldi et al., 2024).

The micro-MPA assumes a constant loading regime and does not account for inter-individual or activity-induced variation in mechanical stimuli. Future work incorporating subject-specific loading profiles or time-varying loading conditions would enhance the physiological fidelity of the model. Such studies could then test whether the current uniaxial loading regime scaled to achieve trabecular bone strains consistent with literature gives results consistent with previous more complex and computationally intensive loading approaches consisting of load estimation of compression and shear along all principal axes, followed by a phase of model relaxation to the loading (Tourolle David Dempster et al., 2021).

The parallelized high-performance computing implementation enabled us to model the entire input scan region with side length 282 × 282 × 264 voxels (voxel size 14 µm) in 8 h for 3 years of denosumab treatment simulation. Thus the limiting factor for these micro-MPA simulations has become the size of the biopsy rather than the computational power requirement. Additional model validation with longitudinal HR-pQCT scans will be key to determining the relevance of trabecular biopsy-based simulations to organ-level results. As the ability of SMI to quantify the rod-vs plate-like characteristics of trabecular bone has been challenged future work could also include investigating a variety of other baseline morphometric parameters that could act as predictors of response to treatment including the ellipsoid factor which better captures the large proportions of concave and saddle curvatures within trabecular bone (Salmon et al., 2015).

The simulations presented in this work suggest that both accumulation of preosteoclasts and osteomorphs play a key role in causing the rapid bone loss following denosumab discontinuation whereas the role of clast-blast coupling and the mechanostat effect is less critical. In all model configurations osteoblast numbers decrease during denosumab treatment by clast-blast coupling and the mean effective strain in the bone decreases due to bone formation at high strain locations. In all configurations of the model, these two latter mechanisms led to a higher RANKL/OPG ratio upon discontinuation and higher sclerostin production by osteocytes, respectively, but those did not match the shape of the clinical denosumab discontinuation bone density curves. Even with RANKL and sclerostin levels not exceeding baseline, the osteoclast precursor accumulation and osteomorphs recycling mechanisms were sufficient to simulate all available clinical data on denosumab discontinuation BMD and serum marker trends. Overall, the micro-MPA model provides a scalable, fast and inexpensive tool to test hypotheses relating to bone mechanobiology and osteoporosis treatment sequences and assist in formulating *in silico* trials to help reduce and refine human clinical trials.

## Materials and methods

### In silico simulations of trabecular bone remodeling using a micro-MPA model

A representative selection of 7 micro-computed tomography (micro-CT) scans of iliac crest biopsies from postmenopausal women matching the demographics (age: 72 ± 5 years) and BV/TV distribution (13.1% ± 4.1%) in the FREEDOM trial for 10 years of denosumab treatment (Dempster et al., 2018) served as input for the baseline model structure. The criteria used to select these biopsies have been thoroughly detailed in previous work (Tourolle David Dempster et al., 2021).

In the micro-MPA model osteoblasts, osteoclasts, osteocytes, MSCs, HSCs, pre-osteoclasts, pre-osteocytes, and lining cells are represented as agents on a voxel-based lattice and are motile and capable of producing or resorbing tissue and signaling molecules. We refer to the model as multiphysics because it couples 1) solid mechanics, 2) diffusion-reaction transport and 3) cell behaviour within the same spatial lattice. Specifically, strain fields are computed via micro-finite element (micro-FE) analysis in ParOSol, a parallel octree solver designed for micro-FE analysis based on micro-CT images (Flaig and Arbenz, 2011) and serve as inputs to cell behavior rules and mechanotransduction signaling, while the signaling molecules RANKL, RANKL-OPG, OPG, sclerostin, TGF-β, and estrogen simultaneously undergo spatial diffusion and biochemical reactions. The pathways linking these cells and cytokines are depicted in Figures 1A–D. Assuming a linear correlation between bone density and local Young’s modulus, ParOSol determines the internal strains which serve as stimulus for the osteocytes and osteoblasts. The micro-MPA model relies on two sets of C++ classes, one set representing the finite element lattice with the concentrations of mineral, osteoid and various cytokines and another C++ superclass and classes defining the behavior and characteristics of the cells/agents. These C++ classes are wrapped in Python and they are called in a Python script where each simulation is initialized and the multiphysics schedule is defined as shown in Figures 1E,F.

Model sensitivity and parameter selection: the current model includes a large number of biological and physical parameters, including cytokine production and decay rates, diffusion coefficients, activation thresholds, and cell-cell interaction rules. Cell numbers and cytokine concentrations in the clinical literature used to initialize the simulations are listed in Table 2 and the full list of model parameters is included in the Supplementary Material in Supplementary Tables S1–S51. To complement this, the initialization procedure is illustrated in Figure 1E. To reduce overfitting, we avoided formal parameter optimization and instead constrained parameters based on literature where available. Where values were not available, parameters were tuned manually within biologically plausible ranges to ensure stable simulations and agreement with trends observed in clinical data. We performed local sensitivity checks by varying individual parameters (e.g., RANKL half-life, osteoblast seeding thresholds) and observing the resulting changes in bone mineral content (BMC) and turnover markers. These checks revealed the model to be particularly sensitive to the dynamics of RANKL and OPG, consistent with the known centrality of the RANK-RANKL-OPG axis in bone remodeling.

**TABLE 2:** Clinical data on cytokine concentrations and cell numbers used to initialize micro-MPA simulations [from (Ledoux et al., 2022)].

Cytokine/cell type	Value	Source, from Ledoux et al. (2022)
RANKL	0.6 pM	Blood serum value
OPG	12.3 pM	Blood serum value
RANKL-OPG	400 pM	Blood serum value
Sclerostin	50 pM	Blood serum value
TGF-β	200 pM	Blood serum value
Estrogen	27.5 pM	Blood serum value for 72 years-old women
Osteoblasts	6.6/mm^2^	Stained 5 µm histology slice
Osteoclasts	0.65/mm^2^ with on average 5 nuclei per osteoclast	TRAP stained 5 µm histology slice
Osteocytes	18,500/mm^3^ BV	Synchrotron analysis of iliac crest biopsy
MSCs	8,000/mm^3^ marrow	Bone marrow supernatant fluid single cell sorting
HSCs	6,000/mm^3^ marrow	Bone marrow supernatant fluid single cell sorting

Mechanical loading conditions: to simulate mechanical stimuli, micro-finite element (micro-FE) analysis was performed using ParOSol on each baseline micro-CT scan. All model parameters related to the mechanics are listed in supplementary content Supplementary Table S5. The loading conditions in previous simulations of 10 years of denosumab (Tourolle David Dempster et al., 2021) were simplified. An axial compressive force was applied to the superior surface, while the inferior surface was fixed. This compressive force was scaled to achieve physiologic strain levels with an effective strain distribution that peaks at 2.5k microstrain in trabecular regions as reported in literature (Al Nazer et al., 2012). The internal strains are computed using a hexahedral FE mesh and assuming a linear correlation between bone density and local Young’s modulus (see Supplementary Table S5). The local internal stresses in the trabecular bone in our simulations were consistent with stresses measured within the cancellous bone in the pelvis (Dalstra and Huiskes, 1995). The local strain fields were used to initialize cell seeding and determine strain-driven signaling (e.g., sclerostin, RANKL/OPG) throughout the simulation.

The center panel in Figure 1E illustrates the initial cell distribution on the trabecular bone surface. To obtain remodelling behavior, the entirety of the 282 × 282 × 264 voxel biopsy scans of isotropic 14 µm voxel resolution were divided into 1000 subregions. Within each of these subregions, if surface voxels were present, the average effective strain on the surface was determined and osteoclasts were seeded at locations on the surface with more than the average effective strain and osteoclasts at locations on the surface with less than the average effective strain if and only if this process resulted in seeding between 6 and 40 osteoblasts and between 4 and 40 osteoclasts within a given subregion. To obtain modelling behavior, osteoblasts and osteoclasts were seeded based on absolute thresholds of effective strain. Osteoblasts were seeded at surface locations where the effective strain was higher than 4000 microstrain and osteoclasts were seeded at surface locations where the effective strain was lower than 200 microstrain. These thresholds were set based on literature reporting physiological loads as 2-3k microstrain, pathological overload above 4k microstrain and rapid bone removal below 200 microstrain (Al Nazer et al., 2012). These thresholds were also used for the strain-dependent production by osteocytes of the signaling molecules sclerostin, RANKL and OPG governing the activation-resorption-formation-quiescence cycle *in silico*.

The final panel in Figure 1E shows the voxel-wise initialization of RANKL concentrations in the example virtual biopsy along with in black the full range of RANKL concentration values reported in literature annotated with their respective references and in the colored bar the range of concentrations of RANKL concentrations over the full course of the simulation.

In the current micro-MPA model, we focus on simulating the behavior and interactions of bone-resorbing and bone-forming cells in a spatially explicit environment, including mechanotransduction and cytokine signaling. While this framework captures key dynamics of bone remodeling under denosumab and placebo conditions, it does not explicitly simulate all contributors to bone mineral density (BMD) changes. For instance, mineralization kinetics are simplified, the amount of new mineral formed added within a voxel over a given timestep is proportional to the difference between the osteoid and the mineral level in that voxel at the start of the timestep and the proportionality factor is such that a voxel saturated with osteoid and containing no mineral will reach 70% mineralization over 1 week. This setup aims to mimic the primary and secondary mineralization patters seen *in vivo* (Lukas et al., 2013; Bala et al., 2010; Ruffoni et al., 2007; Roschger et al., 2008).

### Modifications to micro-MPA model for the physiologic simulation of the biology of both treatment and treatment discontinuation

Osteomorphs were implemented as an additional cell type with a half-life of 6 months, residing in the marrow and moving towards higher RANKL concentrations at an average speed of 14.4 μm/d (McDonald et al., 2021). This cell type provides increased motility and survival of osteoclasts and more rapid differentiation to osteoclasts as RANKL rises. The probability of an osteoclast fissioning to osteomorphs is inversely proportional to its RANK binding site occupancy and the probability of osteomorphs fusing to osteoclasts on the surface is proportional to their RANK binding site occupancy (McDonald et al., 2021). The rate constants for the forward and backward binding reactions of RANKL to osteomorphs and osteoclasts were identical (McDonald et al., 2021). The rate of fission of osteoclasts to osteomorphs when the RANKL level was at 100 ng/mL was 0.6 osteoclasts/hour/(100 × 100 μm^2^).

Upon reassessment of simulation output post inclusion of osteomorphs, updates to the model were made to ensure that the behavior of the system remained biofidelic. Changes were structured around three axes: osteoclast and osteoblast seeding at simulation baseline, the RANK/RANKL/OPG signaling pathway and the TGF-β signaling pathway. These updates also addressed several of the limitations of the 10-year denosumab simulations as outlined in Tourolle David Dempster et al. (2021).

The seeding method for osteoclasts and osteoblasts was adjusted to achieve a closer fit to the distributions of osteoclasts and osteoblasts reported in histology data for postmenopausal osteoporotic patients both in terms of cell numbers (Ledoux et al., 2022) and distribution. The aim was to achieve a mix of modelling- and remodelling-based seeding with osteoclasts at low effective strain locations and osteoclasts at different high effective strain locations in the modelling case and osteoclasts and osteoblasts close together as a basic multicellular unit with more than 3 of each cell type in the remodelling case.

Reassessment of kinetics post inclusion of osteomorphs revealed that the rates of change in mineral density were no longer biofidelic. The behavior of the RANK/RANKL/OPG system was adjusted to obtain rapid bone loss after denosumab discontinuation. The RANKL recovery following denosumab discontinuation was increased by increasing k_off_ for the reversible reaction denosumab + RANKL $\begin{array}{c} k_{on}\\ ⇄\\ k_{off} \end{array}$ RANKLD. The denosumab decay was adjusted downwards to maintain the 26 days half-life of denosumab. The threshold for and magnitude of the effect of RANK binding site occupancy on HSC differentiation to osteoclast precursors on the surface and the threshold for and magnitude of the effect of RANK binding site occupancy on osteomorphs differentiation to osteoclasts were adjusted to obtain a more rapid increase in osteoclast numbers following denosumab discontinuation.

The TGF-β pathway was also updated following the explicit inclusion of osteomorphs to ensure that the rapid bone loss following denosumab discontinuation arrested within 2 years of the final dose, reaching a stable rate of bone loss resembling that was found in the treatment and treatment-naïve control groups from the FREEDOM trial (Dempster et al., 2018). To achieve this, the rate of release of TGF-β from the mineral matrix following resorption by osteoclasts was increased, the diffusivity of TGF-β through the marrow was increased and the threshold for and magnitude of the effect of TGF-β binding site occupancy on differentiation of MSCs to osteoblasts was increased.

The changes outlined above made it possible to obtain long term changes in BMC with denosumab treatment similar to the BMD changes outlined in Dempster et al. (2018) and rates of changes in BMC following denosumab discontinuation similar to the BMD changes outlined in Bone et al. (2018) using a model explicitly incorporating osteomorphs as outlined in McDonald et al. (2021). The parameter updates are summarized in (Supplementary Tables S1–S5).

Each change to the model was required to pass a series of checks and balances. First each parameter change was verified against literature when available (Ledoux et al., 2022). Second, the parameters were required to pass cell number and cytokine concentration balances to have a stable system at equilibrium and limit maximum concentration and cell number rates of change to physiologic maxima for each timestep. Taking osteoblasts as an example, the cell number balances were defined as shown in Equation 1.

$$\frac{dn_{OB}}{dt}=\left(MSC\;\to \;OB\right)+\left(lc\;\to \;OB\right)+\left(OB\;proliferation\right)\;‐\;\left(OB\;\to \;lc\right)\;‐\;\left(OB\;\to \;OCY\right)\;‐\;\left(OB\;apoptosis\right)$$ (1)

Equation 1 describes the rate of change in $n_{OB}$ the number of osteoblasts $\frac{dn_{OB}}{dt}$ as the net sum of the rates of differentiation of MSCs to osteoblasts, lining cells to osteoblasts, osteoblast proliferation minus the rate of differentiation of osteoblasts to lining cells, osteoblasts to osteocytes and the rate of osteoblast apoptosis. Writing each term out into its component model parameters and state variables, the result is Equation 2.

$$\frac{dn_{OB}}{dt}\approx \Gamma_{MSCtoOB}n_{MSC}e_{\bar{SOST},MSCtoOB}e_{\bar{TGF‐\beta },MSCtoOB}+\Gamma_{lctoOB}n_{lc}e_{\bar{SOST},lctoOB}+P_{OB}n_{OB}/7‐\Gamma_{OBtolc}n_{OB}e_{\bar{SOST},OBtolc}\;‐\Gamma_{OBtoOCY}n_{OB}\;‐\frac{A_{OB}n_{OB}}{7}e_{\bar{E},OBapop}$$ (2)

where $\Gamma_{MSCtoOB}$ is the rate of differentiation of MSCs to osteoblasts in % per day, $n_{MSC}$ is the number of MSCs, $e_{\bar{SOST},MSCtoOB}$ is the effect of the current average sclerostin level on the rate of differentiation of MSCs to OBs, $e_{\bar{TGF‐\beta },MSCtoOB}$ is the effect of the current average TGF-β level on the rate of differentiation of MSCs to OBs, $\Gamma_{lctoOB}$ is the rate of differentiation of lining cells to osteoblasts, $n_{lc}$ is the number of lining cells, $e_{\bar{SOST},lctoOB}$ is the effect of the current average sclerostin level on the rate of differentiation of lining cells to OBs, $P_{OB}$ is the proliferation rate of OBs, $\Gamma_{OBtolc}$ is the rate of differentiation of OBs to lining cells, $e_{\bar{SOST},OBtolc}$ is the effect of the current average sclerostin level on the rate of differentiation of OBs to lining cells, $\Gamma_{OBtoOCY}$ is the rate of differentiation of OBs to osteocytes, $A_{OB}$ is the rate of apoptosis of osteoblasts (per week instead of % day) and $e_{\bar{E},OBapop}$ is the effect of the current average estrogen level on the rate of apoptosis of osteoblasts. Cell type-dependent upper and lower limits were set on rates of change in cell numbers at the default pre-menopausal signaling molecule concentration levels (e.g. $\frac{dn_{OB}}{dt}/n_{OB}$ was limited to ±5%/day in simulations of healthy bone remodelling) (Kameo et al., 2020).

### Investigating mechanisms for the rapid bone loss following denosumab discontinuation

*In silico* experiments were designed to test the four mechanistic hypotheses in Figures 1A–D individually and in combination. In all four mechanisms being analyzed, the parameters of the pathway of interest were varied to achieve the best fit possible to clinical data while controlling the other pathways. The constraints on this optimization process were that cell numbers and signaling molecule concentrations would stay within physiologic ranges and that the placebo branch total drop in BMC over 4 years would be between 0.5% and 5%.

To investigate the net influence of each mechanism on bone mineral content BMC trends over time a linear combination model was used that predicts the independent variable, BMC, on the basis of the active mechanisms and their interactions ( PA*PB*PC*PD ), a random intercept (1| patient) and a patient-specific baseline (BL) as shown in Equation 3.

$$BMC\;∼\;PA*PB*PC*PD+\left(1\left|\right.patient\right)+BL$$ (3)

The term BL represents the baseline bone mineral content (BMC) of each patient-specific biopsy at simulation start, while the random intercept (1|patient) accounts for individual-level deviations in BMC trends across simulations. Both terms are matched per biopsy and reflect consistent indexing across the model. This combinatorial linear model, where the BMC is a linear function of 4 categorical variables and all their interactions, may also be written out as seen in Equation 4.

$$BMC=\beta_{A}\;PA+\beta_{B}\;PB+\beta_{C}\;PC+\beta_{D}\;PD+\beta_{AB}\;PA\;PB+\beta_{AC}\;PA\;PC+\beta_{AD}PA\;PD+\beta_{BC}\;PB\;PC+\beta_{BD}PB\;PD+\beta_{CD}\;PC\;PD+\beta_{ABC}\;PA\;PB\;PC+\beta_{ABD}\;PA\;PB\;PD+\beta_{ACD}\;PA\;PC\;PD+\beta_{BCD}\;PB\;PC\;PD+\beta_{ABCD}\;PA\;PB\;PC\;PD+\left(1\left|\;\right.patient\right)+BL$$ (4)

Equation 4 presents the fixed-effects portion of the linear mixed-effects model used to explain simulated BMC changes across different mechanistic configurations. In this equation, βA through βABCD are the fixed-effect coefficients corresponding to each main effect and interaction term between mechanisms PA, PB, PC, and PD. Each P term is a binary indicator variable (0 or 1) denoting whether the corresponding mechanistic pathway - gate-blocking (A), osteomorph recycling (B), clast-blast coupling (C), or mechanostat (D) - was activated in a given simulation. The interaction terms (e.g., PA⋅PB) allow for synergy or antagonism between mechanisms to influence the predicted BMC.

Eight clinical validation outcomes were defined: the relative changes in BMD at 1, 6, 12, 24, 30, 36, 42 and 48 months from baseline in the simulations of 2 years of treatment followed by 2 years without treatment (Fontalis et al., 2020). The linear combination model provides an overview of which mechanisms and mechanism combinations play a role in denosumab discontinuation.

### Parallel processing for high performance computing

Our in-house code employs MPI distributed parallelism and hybrid MPI/OpenMP. We perform large-scale micro-FE analyses up to 25 million elements and 300 million degrees of freedom. Our bone adaptation simulation incorporates an agent-based cell modeling approach, with heterogeneous cell data structures implemented to improve distributed memory parallelism. A novel domain splitting and cell communication method was developed to minimize the required number of MPI operations and significantly increase the speed of the simulations (Kendall et al., 2023; Boaretti et al., 2023). Cell behavior is updated based on the local mechanics computed using ParOSol, a fully parallel micro-finite element code based on an Octree and designed for massively parallel architectures using C++, MPI, and optimized BLAS and LAPACK libraries (Dongarra et al., 1990; Anderson et al., 1990). ParOSol can solve problems that are one order of magnitude larger than available commercial and open-source solutions as it generates meshes directly from image data through voxel-to-element conversion, allowing models with billions of degrees of freedom to be easily produced while substantially reducing memory usage. I/O operations are performed by the Hierarchical Data Format (HDF5) library, such that data files can be read or written in parallel on any architecture that supports HDF5. Preprocessing of patient image data was carried out using scripts from the in-house IFB Framework, containing custom Python modules for 3D image processing. Postprocessing was performed using sequential codes and parallel visualization with ParaView on Eiger (Project s1289, Swiss National Supercomputing Centre, Lugano, Switzerland). A typical simulation requires 8 nodes for the micro-MPA model and 2 nodes for the micro-FE analysis with ParOSol, therefore 10 nodes per analysis. This number of nodes was chosen to optimize the trade-off between computational time and speed-up of the code.

The micro-MPA model has been optimized by implementing several MPI communicators in our code (mpi4py, amgcl, Boost, the standard MPI_comm) as well as a method to pass the mpi4py communicator to the C++ code (Dalcín et al., 2005). In this way, we benefit from the mpi4py interface at high level for standard MPI communications as well as for more advanced low-level computations performed for the data storage and communication with Boost (Siek et al., 2002; Abrahams et al., 2003). Further, the amgcl solver (Demidov et al., 2021; Demidov, 2020; Demidov, 2019) uses the same MPI communicator for solving the diffusion of chemicals in the C++ code through domain splitting. As a result, all our data are stored and computed with domain splitting. In addition, we were able to save the data through the h5py parallel interface, exploiting this parallel implementation (Collette, 2014).

### Morphometric analysis

Simulation outputs were validated against densitometric (percent change in BMD), static morphometric (BV/TV, Tb.N, Tb.Th, Tb.Sp) and dynamic morphometric parameters, e.g. Mineral Apposition Rate (MAR), Mineral Resorption Rate (MRR), Bone Formation Rate (BFR), Bone resorption Rate (BRR), measured in clinical trials according to the guidelines of the American Society for Bone and Mineral research (Parfitt et al., 1987).

## Data Availability

Data including model inputs and all necessary parameterization needed to evaluate the original contributions presented in this study are included in the article/Supplementary Material. Further inquiries can be directed to the corresponding authors.

## Supplementary material

SUPPLEMENTARY FILE 1 This document includes supplementary methods detailing the in silico modeling procedures used in the study, as well as Tables S1 - S5. These tables detail parameterization of cell/signaling molecule initialization, reaction-diffusion decay, behaviour, and model mechanics/timing.
