# Supplementary material for: Digital twin simulations with a micro-multiphysics agent-based model reveal key drivers of bone loss after denosumab discontinuation
Source: Front Bioeng Biotechnol. 2025 Dec 1;13:1652201. doi: 10.3389/fbioe.2025.1652201 (PMC12702982; doi:10.3389/fbioe.2025.1652201)
Supplement: Supplementary file 1 [file Supplementaryfile1.pdf]

Supplementary Materials for

Digital twin simulations with a micro-multiphysics agent-based model reveal key drivers of bone loss after denosumab discontinuation

C. Ledoux, J.J. Kendall, D. Boaretti, R. Müller, C.J. Collins*
*Corresponding author. Email: cjcollins@vt.edu

This PDF file includes:

Supplementary Text

Table S1. Parameters of the micro-MPA model related to initialization of cells and signaling molecules (expansion of Table 2 in the main manuscript)

Table S2. Parameters of the micro-MPA model related to reaction-diffusion-decay of signaling molecules

Table S3. Parameters of the micro-MPA model related to cell behaviour

Table S4. Parameters of the micro-MPA model related to modulation of cell behaviour by signaling molecules

Table S5. Parameters of the micro-MPA model related to mechanics and timeline

Other Supplementaty Materials for this Manuscript include the following:

(available at)

Tourolle, D. (2019). *A Micro-Scale Multiphysics Framework for Fracture Healing and Bone Remodelling* [Doctoral Thesis, ETH Zurich]. ETH Research Collection. https://doi.org/10.3929/ ETHZ-B-000364637.

PhD thesis chapter, *Chapter 4 : In silico fracture healing*, contains details of a preclinical (murine) computational model from which the current manuscript's model was adapted.

Supplementary Methods

S1. In silico model of bone remodeling

The micro-MPA model used in this manuscript is adapted from a model described in detail in Tourolle, D. (2019) (referenced above) and in the main body of Tourolle né Betts et al. 2021 [1]. Major changes made to this model are described in the Methods section of the main manuscript. A brief overview of the behaviour of the cells and signaling molecules in the updated model is included here along with a full list of all parameter values used.

S1.1 Cell behaviour in the micro-MPA model

Osteocytes (OCYs) are modeled as long-lived mechanosensitive cells embedded within the mineralized matrix. They derive from osteoblasts that become entrapped during osteoid mineralization. In the model, osteocytes serve as the primary detectors of mechanical strain and microdamage, regulating downstream bone formation and resorption. They produce the signaling molecules *sclerostin* (an inhibitor of osteoblast activity), *RANKL* (a promoter of osteoclastogenesis), and *OPG* (a decoy receptor for RANKL). These signals mediate the spatial and temporal coordination of remodeling.

Osteoblasts (OBs) arise from mesenchymal stem cells (MSCs) and are explicitly modeled as the agents responsible for osteoid synthesis. These cells populate bone surfaces and secrete matrix proteins such as Type I collagen and alkaline phosphatase. In our framework, their activity is modulated by mechanical strain and osteocytic signals. Osteoblasts may differentiate into osteocytes, become quiescent lining cells, or undergo apoptosis, depending on local environmental cues. Their role in regulating osteoclasts is captured through production of RANKL and OPG.

Osteoclasts (OCLs) are multinucleated, bone-resorbing cells derived from hematopoietic stem cells (HSCs) via the monocyte-macrophage lineage. They are explicitly modeled as agents that degrade mineralized matrix by acidification and enzymatic digestion within resorption lacunae. Osteoclast activation is governed by RANKL-RANK signaling, while OPG serves as an inhibitory control. The model includes osteoclast lifespan, migration to resorption sites, and apoptosis. Estrogen-mediated regulation is included as a parameter influencing survival rates.

Pre-osteoclasts (preOCLs) are mononuclear progenitors that differentiate and fuse into mature osteoclasts. Their recruitment and fusion are driven by local RANKL concentrations and influenced by M-CSF and TGF-β. The differentiation process is regulated by the RANKL/OPG ratio, modeled as a dynamic parameter affected by nearby osteoblasts and osteocytes.

Osteomorphs, are included in our model as a reversible, fusion-competent reservoir of osteoclast precursors. They respond to local RANKL and provide a rapid mechanism for modulating osteoclast number without requiring full differentiation from HSCs [2].

Mesenchymal Stem Cells (MSCs) are multipotent progenitors of osteoblasts in the model. Their proliferation and differentiation are influenced by mechanical cues, TGF-β, and Wnt signaling (modulated by sclerostin). While MSCs can also generate adipocytes and chondrocytes in vivo, only their osteogenic lineage is represented here. MSC dynamics support osteoblast population maintenance and spatial distribution.

Hematopoietic Stem Cells (HSCs) serve as the source of pre-osteoclasts. Their proliferation and lineage commitment are regulated by M-CSF and RANKL in the model, ensuring a continuous supply of osteoclast progenitors. Although HSCs also give rise to immune cells and other lineages, only their role in osteoclastogenesis is included in this framework.

S1.2 Signaling molecules in the micro-MPA model

Receptor Activator of Nuclear factor Kappa-β Ligand (RANKL) is modeled as a key osteoclastogenic cytokine secreted by osteoblasts, osteocytes, and lining cells. It binds to RANK on osteoclast precursors and mature osteoclasts, promoting their differentiation and survival. Its production is regulated in the model by mechanical strain (via osteocytes) and hormonal cues such as PTH. The RANKL–RANK–OPG signaling axis is a core component of our remodeling logic, governing resorption initiation.

Osteoprotegerin (OPG) is implemented as a diffusible, cell-secreted inhibitor of osteoclastogenesis. Produced by osteoblasts, osteocytes, and lining cells, OPG functions as a decoy receptor for RANKL, binding to it and preventing interaction with RANK on osteoclast precursors. Its expression is modeled as a function of estrogen levels and local mechanical conditions. The local RANKL/OPG ratio is a key determinant of osteoclast activation and is updated dynamically within each simulation timestep.

Transforming Growth Factor β (TGF-β) is implemented as a matrix-stored coupling factor released

during osteoclast-mediated resorption. Upon release, it locally stimulates MSC proliferation and differentiation toward the osteoblastic lineage and modulates osteoblast activity, including matrix production and apoptosis resistance. Its dual effect on osteoclastogenesis—indirect inhibition via OPG stimulation and context-dependent promotion of RANK sensitivity—is incorporated into both osteoclast and osteoblast decision modules.

Sclerostin, produced predominantly by osteocytes, is modeled as a local inhibitor of osteoblast activity. It suppresses the Wnt signaling pathway by binding to LRP6 on osteoblasts and MSCs, thereby reducing their proliferation and matrix production. In our framework, sclerostin levels respond inversely to mechanical strain, enabling load-responsive regulation of bone formation. The inhibition of sclerostin under high strain is a key pathway linking mechanical cues to osteogenesis in the model. **LRP6** serves as a coreceptor for Wnt proteins in osteoblasts and MSC. Its activation is necessary for Wnt-mediated bone formation. In our model, LRP6 activity is dynamically modulated by sclerostin binding, and its state determines the strength of Wnt signaling, influencing cell proliferation and osteoblastogenesis.

Estrogen's effects on the skeleton are mediated by a multitude of pathways in vivo [3]. Applying Occam's razor we have chosen in our model to include only that estrogen promotes osteoblast and osteocyte survival while inducing apoptosis in osteoclasts all by direct binding to these cell types. In estrogen-deficient conditions, the model reproduces increased resorptive activity and net bone loss.

Osteoid, in our model, represents the unmineralized organic matrix synthesized by active osteoblasts. It is primarily composed of Type I collagen and various noncollagenous proteins, forming a scaffold for subsequent mineralization. Osteoid thickness evolves as a function of osteoblast secretion rates and is essential for capturing early phases of bone matrix formation. Its presence influences osteoblast-to-osteocyte transition, as embedded osteoblasts begin differentiation once entrapped within osteoid layers. We model osteoid not only as a precursor to mineralized matrix but also as a regulator of cellular dynamics, affecting osteoclast resorption patterns and the spatial distribution of mechanical cues.

Mineralization is modeled as a delayed, enzyme-mediated process that converts osteoid into mineralized bone tissue (mineral). Initiated by osteoblast-secreted vesicles containing alkaline phosphatase, mineralization requires sufficient local concentrations of calcium and phosphate ions. In the model, the local mineral concentration increases over each timestep at a rate directly proportional to the local difference between osteoid and mineral concentration at the start of the timestep. Mineral concentration is directly proportional to matrix stiffness and also determines whether matrix is targetable by osteoclasts, which preferentially resorb mineralized tissue.

Supplementary Tables

Table S1: Parameters of the micro-MPA model related to initialization of cells and signaling molecules (expansion of Table 2 in the main manuscript) [4]

**Symbol**	**Description**	**Value**	**Unit**
$[\text{RANKL}]_{t=0}$	RANKL concentration at baseline	0.6	pM
$[\text{OPG}]_{t=0}$	OPG concentration at baseline	12.3	pM
$[\text{RANKLOPG}]_{t=0}$	RANKL–OPG concentration at baseline	400	pM
$[\text{SOST}]_{t=0}$	Sclerostin concentration at baseline	50	pM
$[\text{TGF-}\beta]_{t=0}$	TGF-β concentration at baseline	200	pM
$[E]_{t=0,\text{PMO}}$	Estrogen concentration at baseline in post-menopausal conditions	27.5	pM
$[E]_{t=0,\text{pre}}$	Estrogen concentration at baseline in pre-menopausal conditions	100	pM
n_{OB}	Number density of OBs on bone surface at baseline	6.6	$/\,\text{mm}^2$
n_{OC}	Number density of multinucleated osteoclasts on bone surface at baseline with median 5 nuclei per osteoclast (1 nucleus occupies 1 voxel in the micro-MPA model)	0.65	$/\,\text{mm}^2$
n_{preOCL}	Number density of preOCLs on bone surface at baseline	2.32	$/\,\text{mm}^2$
n_{LC}	Number density of LCs on bone surface at baseline	3	$/\,\text{mm}^2$
n_{OCY}	Number density of OCYs within bone at baseline	18,500	$/\,\text{mm}^3$
n_{MSCs}	Number density of MSCs in marrow at baseline	8,000	$/\,\text{mm}^3$
n_{HSCs}	Number density of HSCs in marrow at baseline	6,000	$/\,\text{mm}^3$

Table S2: Parameters of the micro-MPA model related to reaction-diffusion-decay of signaling molecules

Symbol	Description	Value	Unit
$k_{\mathrm{on,RANK}}$	Rate of binding of RANKL onto its receptor RANK	0.0144	$\mathrm{M}^{-1}\,\mathrm{s}^{-1}$
$k_{\mathrm{off,RANK}}$	Rate of unbinding of RANKL from its receptor RANK	0.107	s^{-1}
$k_{\mathrm{on,RANKLOPG}}$	Rate of forward reaction between OPG and RANKL	0.00124	$\mathrm{M}^{-1}\,\mathrm{s}^{-1}$
$k_{\mathrm{off,RANKLOPG}}$	Rate of backward reaction between OPG and RANKL	0.00503	s^{-1}
$k_{\mathrm{on,E}}$	Rate of binding of estrogen onto its receptor	0.002	$\mathrm{M}^{-1}\,\mathrm{s}^{-1}$
$k_{\mathrm{off,E}}$	Rate of unbinding of estrogen from its receptor	0.0001	s^{-1}
$k_{\mathrm{on,LRP6}}$	Rate of binding of sclerostin onto its receptor LRP6	0.002	$\mathrm{M}^{-1}\,\mathrm{s}^{-1}$
$k_{\mathrm{off,LRP6}}$	Rate of unbinding of sclerostin from its receptor LRP6	0.0001	s^{-1}
$k_{\mathrm{on,TGF}\text{-}\beta}$	Rate of binding of TGF-$\boldsymbol{\beta}$ onto its receptor	0.002	$\mathrm{M}^{-1}\,\mathrm{s}^{-1}$
$k_{\mathrm{off,TGF}\text{-}\beta}$	Rate of unbinding of TGF-$\boldsymbol{\beta}$ from its receptor	0.0001	s^{-1}
D_{RANKL}	Diffusion rate of RANKL	1	cm^2/s
D_{OPG}	Diffusion rate of OPG	7	cm^2/s
D_{RANKLOPG}	Diffusion rate of RANKL–OPG	107	cm^2/s
D_{SOST}	Diffusion rate of Sclerostin	107	cm^2/s
$D_{\mathrm{TGF}\text{-}\beta}$	Diffusion rate of TGF-$\boldsymbol{\beta}$	0.2	cm^2/s
D_E	Diffusion rate of estrogen	107	cm^2/s
$\mathrm{dec}_{\mathrm{RANKL}}$	Decay/evacuation rate of RANKL	0.02	/day
$\mathrm{dec}_{\mathrm{OPG}}$	Decay/evacuation rate of OPG	0.02	/day
$\mathrm{dec}_{\mathrm{RANKLOPG}}$	Decay/evacuation rate of RANKLOPG	0.02	/day
$\mathrm{dec}_{\mathrm{SOST}}$	Decay/evacuation rate of Sclerostin	0.02	/day
$\mathrm{dec}_{\mathrm{TGF}\text{-}\beta}$	Decay/evacuation rate of TGF-$\boldsymbol{\beta}$	0.02	/day
dec_E	Decay/evacuation rate of estrogen	0.001	/day

Table S3: Parameters of the micro-MPA model related to cell behaviour

Symbol	Description	Value	Unit
A_{OCL}	Max apoptosis rate of OCL	2.31	%/week
A_{OB}	Max apoptosis rate of OBs	2.02	%/week
A_{OCY}	Max apoptosis rate of OCYs	0.077	%/week
A_{preOCL}	Max apoptosis rate of preOCLs	0.32	%/week
A_{HSC}	Max apoptosis rate of HSCs	3.02	%/week
A_{MSC}	Max apoptosis rate of MSCs	3.02	%/week
A_{LC}	Max apoptosis rate of LCs	0.20	%/week
P_{OCL}	Max proliferation rate of OCLs	0	%/week
P_{OB}	Max proliferation rate of OBs	2.02	%/week
P_{OCY}	Max proliferation rate of OCYs	0	%/week
P_{preOCL}	Max proliferation rate of preOCLs	0	%/week
P_{HSC}	Max proliferation rate of HSCs	3.76	%/week
P_{MSC}	Max proliferation rate of MSCs	3.76	%/week
P_{LC}	Max proliferation rate of LCs	0	%/week
$\mathrm{prod}_{\mathrm{RANKL,OCY}}$	Max RANKL production by OCY	2	pM/day
$\mathrm{prod}_{\mathrm{sclerostin,OCY}}$	Max sclerostin production by OCY	2.3	pM/day
$\mathrm{prod}_{\mathrm{OPG,OCY}}$	Max OPG production by OCY	4	pM/day
$\mathrm{prod}_{\mathrm{OPG,OB}}$	Max OPG production by OB	4	pM/day
$\mathrm{prod}_{\mathrm{osteoid,OB}}$	Max osteoid production by OB	5	pM/day
$\mathrm{prod}_{\mathrm{TGF}\text{-}\beta\mathrm{,OCL}}$	Max TGF-β release from matrix by OCL	5	pM/day
$\mathrm{prod}_{\mathrm{osteoid,OCL}}$	Max osteoid resorption level by OCL	-5	pM/day
$\mathrm{prod}_{\mathrm{mineral,OCL}}$	Max mineral resorption level by OCL	-5	pM/day
$\mathrm{prod}_{\mathrm{OPG,LC}}$	Max OPG production level by LCs	4	pM/day
$\Gamma_{\mathrm{HSC}\to\mathrm{pre\text{-}OCL}}$	Max differentiation rate HSC to pre-OCL	0.5	%/day
$\Gamma_{\mathrm{pre\text{-}OCL}\to\mathrm{OCL}}$	Max differentiation rate pre-OCL to OCL	0.5	%/day
$\Gamma_{\mathrm{OCL}\to\mathrm{osteomorph}}$	Max differentiation rate OCL to osteomorph	5	%/day
$\Gamma_{\mathrm{osteomorph}\to\mathrm{OCL}}$	Max differentiation rate osteomorph to OCL	5	%/day
$\Gamma_{\mathrm{MSC}\to\mathrm{OB}}$	Max differentiation rate MSC to OB	0.5	%/day
$\Gamma_{\mathrm{OB}\to\mathrm{LC}}$	Max differentiation rate OB to LC	0.02	%/day
$\Gamma_{\mathrm{LC}\to\mathrm{OB}}$	Max differentiation rate LC to OB	0.02	%/day
$\Gamma_{\mathrm{OB}\to\mathrm{OCY}}$	Max differentiation rate OB to OCY	0.05	%/day
v_{OCL}	Max speed of OCLs	84.0	μm/day
v_{OB}	Max speed of OBs	84.0	μm/day
v_{preOCL}	Max speed of preOCLs	84.0	μm/day
v_{MSC}	Max speed of MSCs	84.0	μm/day
v_{HSC}	Max speed of HSCs	84.0	μm/day

Table S4: Parameters of the micro-MPA model related to modulation of cell behaviour by signaling molecules

Symbol	Description	Value	Unit
$c_{E,\mathrm{OCLapop}}$	Estrogen effect on OCL apoptosis	2	Factor
$c_{E,\mathrm{OBapop}}$	Estrogen effect on OB apoptosis	0.13	Factor
$c_{E,\mathrm{OCYapop}}$	Estrogen effect on OCY apoptosis	0.13	Factor
$c_{\mathrm{RANK,HSC}\to\mathrm{preOCL}}$	RANK effect on HSC to preOCL	2	Factor
$c_{\mathrm{RANK,preOCL}\to\mathrm{OCL}}$	RANK effect on preOCL to OCL	2	Factor
$c_{\mathrm{RANK,HSCprolif}}$	RANK effect on HSC proliferation	2	Factor
$c_{\mathrm{RANK,preOCLapop}}$	RANK effect on preOCL apoptosis	0.01	Factor
$c_{\mathrm{RANK,OCLapop}}$	RANK effect on OCL apoptosis	0.01	Factor
$c_{\mathrm{LRP6,MSC}\to\mathrm{OB}}$	Sclerostin/LRP6 effect on MSC to OB	6	Factor
$c_{\mathrm{LRP6,OB}\to\mathrm{LC}}$	Sclerostin/LRP6 effect on OB to LC	1.2	Factor
$c_{\mathrm{LRP6,LC}\to\mathrm{OB}}$	Sclerostin/LRP6 effect on LC to OB	0.8	Factor
$c_{\mathrm{TGF}\text{-}\beta,\mathrm{MSC}\to\mathrm{OB}}$	TGF-β effect on MSC to OB	6	Factor
$c_{\mathrm{TGF}\text{-}\beta,\mathrm{MSCprolif}}$	TGF-β effect on MSC proliferation	6	Factor
$bs_{\mathrm{RANK,OCL}}$	Number of RANK binding sites on OCL	8000	bs/cell
$O_{\mathrm{RANK,OCL}}$	% occupancy of RANK binding sites on OCL at baseline	100	%
$bs_{\mathrm{RANK,preOCL}}$	Number of RANK binding sites on preOCL	8000	bs/cell
$O_{\mathrm{RANK,preOCL}}$	% occupancy of RANK binding sites on preOCL at baseline	100	%
$bs_{\mathrm{RANK,HSC}}$	Number of RANK binding sites on HSCs	8000	bs/cell
$O_{\mathrm{RANK,HSC}}$	% occupancy of RANK binding sites on HSCs at baseline	100	%
$bs_{\mathrm{LRP6,OB}}$	Number of LRP6 binding sites on OBs	8000	bs/cell
$O_{\mathrm{LRP6,OB}}$	% occupancy of LRP6 binding sites on OBs at baseline	100	%
$bs_{\mathrm{LRP6,MSC}}$	Number of LRP6 binding sites on MSCs	8000	bs/cell
$O_{\mathrm{LRP6,MSC}}$	% occupancy of LRP6 binding sites on MSCs at baseline	100	%
$bs_{\mathrm{LRP6,LC}}$	Number of LRP6 binding sites on lining cells	8000	bs/cell
$O_{\mathrm{LRP6,LC}}$	% occupancy of LRP6 binding sites on LCs at baseline	100	%
$bs_{\mathrm{LRP6,OCY}}$	Number of LRP6 binding sites on OCYs	8000	bs/cell
$O_{\mathrm{LRP6,OCY}}$	% occupancy of LRP6 binding sites on OCYs at baseline	100	%
$bs_{\mathrm{TGF}\text{-}\beta,\mathrm{OB}}$	Number of TGF-β binding sites on OBs	8000	bs/cell
$O_{\mathrm{TGF}\text{-}\beta,\mathrm{OB}}$	% occupancy of TGF-β binding sites on OBs at baseline	100	%
$bs_{\mathrm{TGF}\text{-}\beta,\mathrm{MSC}}$	Number of TGF-β binding sites on MSCs	8000	bs/cell
$O_{\mathrm{TGF}\text{-}\beta,\mathrm{MSC}}$	% occupancy of TGF-β binding sites on MSCs at baseline	100	%
$bs_{E,\mathrm{OB}}$	Number of estrogen binding sites on OBs	8000	bs/cell
$O_{E,\mathrm{OB}}$	% occupancy of estrogen binding sites on OBs at baseline	100	%
$bs_{E,\mathrm{OCL}}$	Number of estrogen binding sites on OCLs	8000	bs/cell
$O_{E,\mathrm{OCL}}$	% occupancy of estrogen binding sites on OCLs at baseline	100	%

Table S5: Parameters of the micro-MPA model related to mechanics and timeline

Symbol	Description	Value	Unit
E_{bone}	Young's modulus of bone	10	GPa
E_{marrow}	Young's modulus of marrow	3	MPa
$\text{rate}_{\text{mineral}}$	Mineralisation rate of osteoid	70	%/week
F_z	Uniaxial compression force	1.5	N
$\varepsilon^{\text{eff}}_{\text{osteogenic}}$	Threshold effective strain for osteogenic stimulus	0.002	strain
$\Delta t_{\text{cell behaviour}}$	Timestep for cell behaviour	4	hours
Δt_{rd}	Timestep for reaction–diffusion of cytokines	0.33	hours
Δt_{mech}	Timestep for calculation of mechanical signal	24	hours

References

[1] Duncan C Tourolle et al. "Ten-year simulation of the effects of denosumab on bone remodeling in human biopsies". In: *JBMR Plus* 5.6 (2021), e10494.

[2] Michelle M McDonald et al. "Osteoclasts recycle via osteomorphs during RANKL-stimulated bone resorption". In: *Cell* 184.5 (2021), 1330–1347.e13.

[3] Sundeep Khosla, Merry Jo Oursler, and David G Monroe. "Estrogen and the skeleton". In: *Trends Endocrinol. Metab.* 23.11 (2012), pp. 576–581.

[4] Charles Ledoux et al. "Clinical data for parametrization of in silico bone models incorporating cell-cytokine dynamics: A systematic review of literature". In: *Front. Bioeng. Biotechnol.* 10 (2022), p. 901720.
